# The Additive Effects of Curcumin Supplementation in Addition to an Anti‐Inflammatory Diet on Inflammatory Indices in Patients With Hashimoto's Thyroiditis (HT): A Double Blind Randomized Controlled Clinical Trial

**DOI:** 10.1002/fsn3.71572

**Published:** 2026-03-11

**Authors:** Fatemeh Bourbour, Behnam Mahdavi, Niayesh Naghshi, Zahra Yari, Seyedsina Moghimnejad hosseini, Saeid Kalbasi, Golbon Sohrab

**Affiliations:** ^1^ Department of Clinical Nutrition and Dietetics, Faculty of Nutrition Sciences and Food Technology, National Nutrition and Food Technology Research Institute Shahid Beheshti University of Medical Sciences Tehran Iran; ^2^ Saeed Pathobiology and Genetics Lab Tehran Iran; ^3^ Department of Nutrition Research, National Nutrition and Food Technology Research Institute and Faculty of Nutrition Sciences and Food Technology Shahid Beheshti University of Medical Sciences Tehran Iran; ^4^ Faculty of Medicine Semmelweis University Budapest Hungary; ^5^ Internal Medicine Ward, Endocrinology and Metabolism Section Shahid Beheshti University of Medical Sciences, Iranian Diabetes Society President Tehran Iran

**Keywords:** anti‐inflammatory diet, curcumin, Hashimoto's thyroiditis, inflammation

## Abstract

Hashimoto's thyroiditis (HT) is a chronic autoimmune condition characterized by persistent thyroid inflammation. Patients with HT may benefit from anti‐inflammatory strategies. This study was designed to examine the combined impact of an anti‐inflammatory diet and curcumin supplementation on inflammatory markers in individuals with HT. In this randomized controlled clinical trial, 57 individuals diagnosed with Hashimoto's thyroiditis (HT) in Tehran, Iran, were recruited and randomly allocated to two intervention arms. One group received an anti‐inflammatory diet in conjunction with 1500 mg of curcumin daily, while the other group followed the same diet but was administered a placebo. Inflammatory biomarkers including high‐sensitivity C‐reactive protein (hs‐CRP), interleukin‐6 (IL‐6), and nuclear factor kappa B (NF‐κB) were measured at the start of the study and again following a 12‐week supplementation period to assess the additive effect of curcumin. Curcumin supplementation resulted in a reduction in IL‐6 (−5.28 ± 9.75, *p* = 0.009). Additionally, it decreased hs‐CRP levels in patients with HT, with a mean reduction of −1.17 ± 5.70 mg/L, whereas the placebo group exhibited an increase of +1.53 ± 3.33 mg/L (*p* = 0.305). Also, curcumin supplementation significantly reduced NF‐κB levels in patients with HT (−0.09 ± 0.22 vs. 0.03 ± 0.15, *p* = 0.019), while the placebo group showed a slight increase (0.65 ± 0.16 to 0.68 ± 0.13). Curcumin, by reducing inflammation, can be effective as an adjunctive treatment alongside an anti‐inflammatory diet in individuals with HT. Additional studies are warranted.

Name of the Registry: National Nutrition and Food Technology Research Institute. Trial Registration Number: NCT0597586. Date of Registration: 2023‐08‐04. URL of Trial Registry Record: https://www.clinicaltrials.gov/study/NCT05975866?term = NCT05975866&rank = 1.

## Graphical abstract

Curcumin supplementation resulted in a reduction in IL‐6. Additionally, it decreased hs‐CRP levels in patients with HT, with a mean reduction of whereas the placebo group exhibited an increase of +1.53 ± 3.33 mg/L (*p* = 0.305). Also, curcumin supplementation significantly reduced NF‐κB levels in patients with HT, while the placebo group showed a slight increase. Curcumin, by reducing inflammation, can be effective as an adjunctive treatment alongside an anti‐inflammatory diet in individuals with HT.

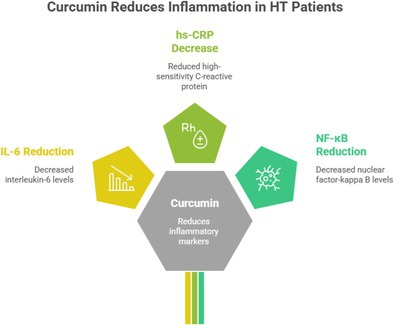

## 1 Introduction

Hashimoto's thyroiditis (HT), also known as chronic lymphocytic thyroiditis, is the most common autoimmune disorder affecting the thyroid gland, with a worldwide prevalence of 10%–12% among adults (Ajjan and Weetman 2015), (Attard and Vella 2018). The condition is marked by the presence of antithyroid antibodies and the activation of T lymphocytes, which contribute to chronic inflammation and gradual fibrotic changes within the thyroid gland, ultimately impairing its function (Wrońska et al. 2024). Chronic fatigue, mood fluctuations, and cardiovascular or gastrointestinal disorders are among the symptoms of this autoimmune disease, which impairs thyroid function (Caturegli et al. 2014). HT is the result of a multifaceted interaction between genetic predisposition, particularly immune and thyroid‐related genes, and environmental factors, including iodine excess, selenium and iron deficiency, hygiene, and gut microbiome alterations, all contributing to immune dysregulation and thyroid autoimmunity (Merrill and Minucci 2018; Tomer 2010; Knezevic et al. 2020; Kivity et al. 2011; Huwiler et al. 2024; Garofalo et al. 2023).

Environmental factors contribute 20%–30%, while genetic factors are responsible for 70%–80% of the risk associated with the development of HT (Wiersinga 2016). The level of thyroid hormones was reported to be related to the level of body fat and inflammatory factors (Lei et al. 2019; Popławska‐Kita et al. 2014; Hu and Rayman 2017). Dietary modifications, particularly an anti‐inflammatory diet, may help prevent the progression of HT (Danailova et al. 2022). An anti‐inflammatory diet that emphasizes whole, unprocessed foods that are rich in fruits, vegetables, and sources of omega‐3 polyunsaturated fatty acids (e.g., fatty fish and nuts), in conjunction with a low intake of refined sugars, has been associated with reduced systemic inflammation (Gholamalizadeh et al. 2022; Vahid et al. 2020) and may assist in modulating the autoimmune response in HT, as demonstrated by previous studies (Kaličanin et al. 2020; Ruggeri et al. 2023).

Curcumin, or diferuloylmethane, is the principal bioactive polyphenol present in turmeric ( *Curcuma longa* ). It has garnered significant scientific and medical attention over the past few decades as a result of its extensive array of potential health benefits. Notably, curcumin has demonstrated promising therapeutic properties, including strong anti‐inflammatory, anti‐diabetic, anti‐cancer, and anti‐aging effects (Akter et al. 2019; Kocaadam and Şanlier 2017; Abd El‐Hack et al. 2021; Sharma et al. 2005). Curcumin has been shown to protect thyroid function by reducing oxidative stress, modulating inflammatory pathways, and preserving histological architecture of the gland (Abdelaleem et al. 2018; Dong et al. 2022). A study indicated that a 10‐week intake of 1500 mg of curcumin significantly reduces the average concentration of C‐reactive protein (CRP), an inflammatory marker (Adibian et al. 2019). For example, Papież (2023) reported that curcumin supplementation improved thyroid morphology and cytochrome c oxidase activity in rats with propylthiouracil‐induced hypothyroidism (Papież 2023).

Studies on the effectiveness of curcumin supplements in reducing systemic inflammation have yielded contradictory results, with one study reporting that an 80 mg curcumin supplement taken for 8 weeks did not significantly decrease CRP concentration (Abd El‐Hack et al. 2021; Bateni et al. 2022; Kabodan et al. 2018). These discrepancies may be explained by differences in dosage, duration of intervention, study design, and characteristics of the study population. Such inconsistencies highlight the need for further trials with standardized protocols. As evidence indicates, curcumin supplementation and adherence to an anti‐inflammatory diet may contribute to reducing inflammatory responses in patients with Hashimoto's thyroiditis (Danailova et al. 2022; Ihnatowicz et al. 2020; Rayman 2019). This study is the first to evaluate the combined effects of curcumin supplementation and an anti‐inflammatory diet in patients with Hashimoto's thyroiditis. Our findings suggest that this 12‐week intervention may help reduce inflammatory markers, underscoring the relevance of curcumin as a potential adjunctive therapy.

## 2 Methods and Materials

### 2.1 Study Design and Participants

This randomized controlled clinical trial was carried out during 2023–2024 on individuals newly diagnosed with HT who were referred for the first time to healthcare centers associated with Shahid Beheshti University of Medical Sciences in Tehran, Iran. The study was officially registered with the Iranian Registry of Clinical Trials under the identification number NCT05975866. Eligible patients, as determined by laboratory results and endocrinologist approval, were invited to participate. Inclusion criteria included a willingness to participate, a confirmed diagnosis of HT by an endocrinologist, age between 20 and 70 years, body mass index (BMI) between 18.5 and 35 kg/m^2^, Participants were excluded if they failed to consume more than 90% of the provided supplements, as adherence below this threshold is generally considered inadequate in nutritional intervention trials (Lichtenstein et al. 2021). Additional exclusion criteria included the use of glucocorticoids, non‐steroidal anti‐inflammatory drugs, or carvedilol. Patients were also excluded if they were pregnant or breastfeeding, had renal disorders, followed non‐standard weight‐loss regimens within 3 months prior to enrollment, or used weight‐loss medications, fiber supplements, or other mineral, herbal, or vitamin supplements. Written consent was obtained at enrollment from all participants. Sample size was calculated based on the dependent variable, plasma hs‐CRP, ensuring a minimum detectable difference of 3 mg/L between groups with 95% confidence (α = 0.05) and 80% power (β = 0.20). The number of samples for each of the groups was estimated to be 23 patients, and considering the possible loss to follow‐up, 31 patients were considered in each group.

**FIGURE 1:**
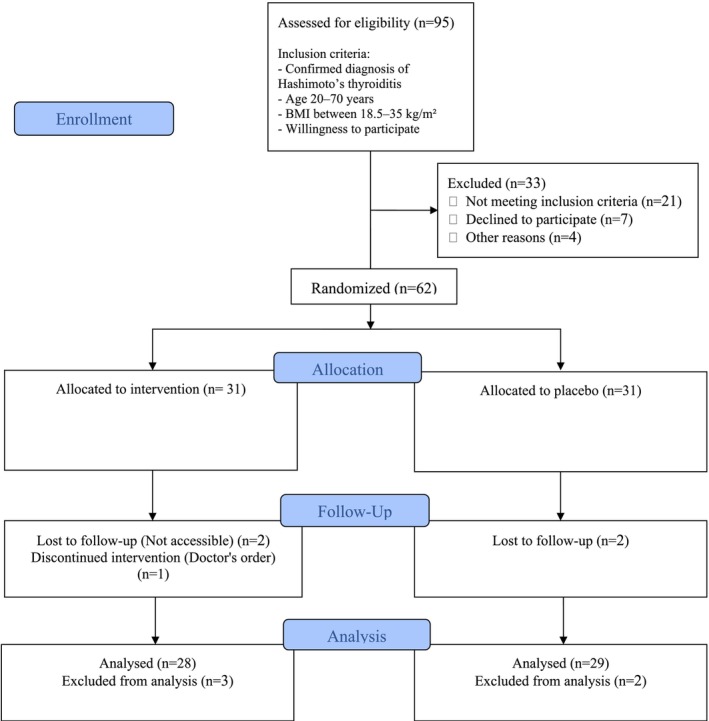
Flowchart of the study participants.

**FIGURE 2:**
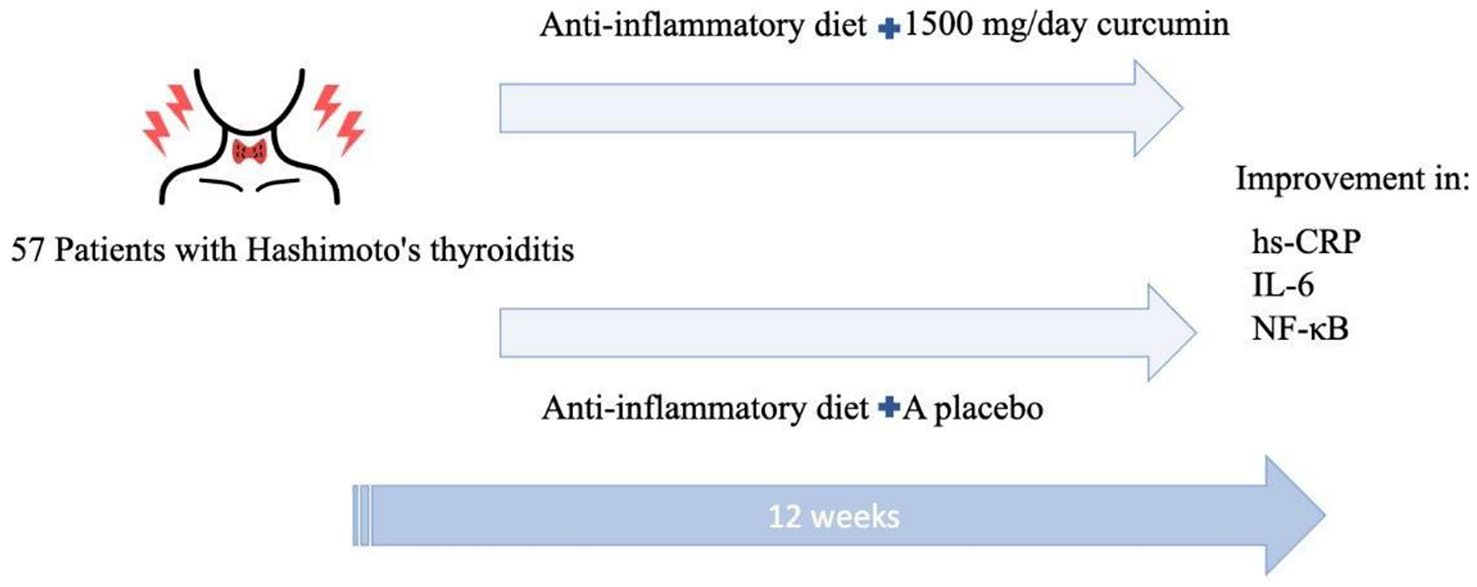
The effects of an anti‐inflammatory diet and curcumin supplementation on patients with Hashimoto's thyroiditis.

Initially, sixty‐two eligible participants, meeting the inclusion criteria, were randomly selected and assigned to intervention (1500 mg curcumin daily: three 500 mg capsules, with each main meal) (*n* = 31) or control (placebo) (*n* = 31) group (three 500 mg placebo capsules containing microcrystalline cellulose, taken with each main meal) (Adibian et al. 2019). Of which, 3 were excluded due to unwillingness to continue cooperation, 1 due to a doctor's prescription not to take curcumin supplements, and 1 due to traveling and unavailability for post‐intervention measurements. Finally, the analysis was performed on 57 people, including 28 in the intervention group and 29 in the control group (Figures 1 and 2).

Curcumin and placebo capsules were provided by Arjuna Natural Extracts Ltd. (Kerala, India). Each curcumin capsule contained a total of 440 mg of curcuminoids, including 347 mg of curcumin, 84 mg of desmethoxycurcumin, and 9 mg of bis‐desmethoxycurcumin, along with 38 mg of turmeric oil. The placebo capsules were identical in appearance, shape, and color to ensure blinding and were composed of 444 mg of cooked rice flour to match the active supplement's physical characteristics. Random allocation was conducted by an independent third party using stratified block randomization to ensure balance between participants in terms of levothyroxine intake and gender. The randomization sequence was prepared in advance by an individual not involved in the research team. Each participant was assigned a sealed envelope containing their group allocation, which was opened sequentially at the time of enrollment. This procedure guaranteed concealment of allocation. Both researchers and participants remained blinded throughout the study, as the curcumin and placebo capsules were identical in appearance, shape, and color. As it is impossible to accomplish the objectives of an anti‐inflammatory diet by consuming a single type of food or adhering to its principles in a single meal, it must be implemented in a consistent and coherent manner within a diet plan (Stromsnes et al. 2021), we recommended an anti‐inflammatory diet. Participants were advised to follow an anti‐inflammatory dietary regimen emphasizing whole, unprocessed foods rich in fruits, vegetables, legumes, whole grains, and sources of omega‐3 fatty acids such as fatty fish and nuts. Intake of refined sugars, processed meats, and trans fats was discouraged. Compliance with the anti‐inflammatory diet was monitored through weekly phone calls, capsule counts, and 3‐day dietary recalls conducted at baseline and at the end of the study. Dietary recalls covered two weekdays and one weekend day to capture variability in eating habits. Trained nutritionists conducted face‐to‐face interviews using standardized portion‐size photographs and household measures to improve accuracy. Adherence was evaluated by comparing reported intake against the recommended dietary guidelines. Participants were considered compliant if they adhered to at least 80% of the prescribed dietary recommendations, a threshold commonly used in nutritional intervention trials to ensure validity of dietary adherence.

The randomization sequence was generated in advance by an independent staff member from the clinical trial unit who was not involved in patient recruitment or data collection. This individual prepared sealed, opaque envelopes containing the group assignments according to the stratified block randomization schedule. The envelopes were numbered sequentially and opened only at the time of enrollment, ensuring allocation concealment and preventing any influence from the investigators. Weekly phone calls were made to ensure adherence and address any patient concerns.

### 2.2 Data Gathering

Dietary intake and physical activity levels were evaluated through in‐person interviews. Dietary intake was assessed using a 3 day food recall covering two weekdays and one weekend day to account for variations in dietary habits. Trained nutritionists conducted face‐to‐face interviews with participants, using standardized portion‐size photographs and household measures to estimate food quantities. The recalls were performed at baseline and after the 12 week intervention, with additional clarifications obtained by telephone when needed. All interviewers had formal training in nutrition sciences and prior experience in dietary assessment, ensuring accuracy and consistency in data collection. Physical activity was measured with the International Physical Activity Questionnaire (IPAQ) (Moghaddam et al. 2012). Anthropometric parameters, including body weight, height, waist circumference, and hip circumference, were measured at baseline and after the 12‐week intervention. Weight was measured using a digital electronic scale (Seca 707; Seca, Hanover, MD) with a precision of 100 g, and height was assessed using a stadiometer equipped with a tape meter. These standardized procedures ensured accurate and consistent evaluation of participants' body composition throughout the study period. Hip circumference was measured at the widest area over the greater trochanters, whereas waist circumference was assessed at the midpoint between the lower margin of the last palpable rib and the upper border of the iliac crest. A non‐elastic, stretch‐resistant tape was used to ensure precision and consistency in anthropometric data collection.

### 2.3 Biochemical Measurements

At baseline and after the 12‐week intervention, a 10 mL venous blood sample was obtained following a fasting period of 12–14 h. Plasma was subsequently separated and stored at −80°C for biochemical evaluations. The concentration of serum tumor necrosis factor‐α (TNF‐α) was determined using a commercially available enzyme‐linked immunosorbent assay (ELISA) kit, ensuring specificity and accuracy in the measurement process. Interleukin‐6 levels were assessed using an ELISA human interleukin‐6 kit (LDN Company, Berlin, Germany). The hs‐CRP was also assessed using a Biopars ELISA kit (Afaq Alborz Teb Company, Tehran, Iran). The measurement of NFKP‐B in peripheral blood mononuclear cells (PBMCs) was conducted utilizing an ELISA kit (Zellbio, Germany). Blood samples (10 mL) were obtained at the commencement and conclusion of the twelfth week of the study. Two milliliters were allocated to microtubes containing EDTA, while the remaining eight milliliters were utilized for plasma and PBMC separation. Blood was centrifuged at 4000 rpm for 10 min, resulting in the separation of plasma. Peripheral blood mononuclear cells (PBMCs) were extracted using the Ficoll density gradient technique. The Buffy Coat was diluted with PBS in a 3:1 ratio and placed over Ficoll, thereafter subjected to centrifugation at 2300 rpm for 20 min. PBMCs were isolated, rinsed twice with PBS, and preserved at −80°C until analysis.

### 2.4 Statistical Analyses

SPSS version 24 (SPSS Inc., Chicago, IL, USA) was employed to conduct statistical analyses. Normality of the data distribution was evaluated using the Kolmogorov–Smirnov test and all variables met the normality assumptions; therefore, parametric tests were applied throughout the analyses. Mean ± standard deviation and frequency (percentage) are the formats in which quantitative and qualitative data are presented, respectively. To examine changes within each group over the course of the study, the paired‐samples *t*‐test was applied. Furthermore, for the comparison of qualitative variables such as gender between the two groups, the chi‐square test was conducted to determine any significant associations. To account for confounding factors, such as the baseline value of the variable, analysis of covariance (ANCOVA) was implemented. *p*‐value < 0.05 was considered statistically significant.

## 3 Results

Both groups were similar in terms of gender distribution, age, disease duration, medication use, marital status, smoking, hookah and alcohol consumption, and physical activity levels (Table 1). The intervention group comprised predominantly females (89.2%), with a mean age of 42.14 years, and the control group had similar demographics. Also, no significant differences were seen between the groups regarding the duration of HT (7.22 ± 5.5 years vs. 6.2 ± 7.19 years, *p* = 0.378) and use of thyroid‐related medication (20 vs. 21, *p* = 0.934). Overall, the groups were well‐matched at baseline regarding different variables.

**TABLE 1:** General characteristics of patients with Hashimoto's thyroiditis in curcumin and placebo groups.

Variables		Curcumin group (*N* = 28)	Placebo group (*N* = 29)	*p*‐value
Gender	Female	25 (89.3%)	25 (86.2%)	0.723
	Male	3 (10.7%)	4 (13.7%)	
Age (y)	Total	42.14 ± 12.63	44.59 ± 9.88	0.419
Duration of Hashimoto's thyroiditis (y)	Total	7.22 ± 5.5	6.2 ± 7.19	0.378
Use of Thyroid‐related medications	Yes	20 (71.4%)	21 (72.4%)	0.934
	No	8 (28.6%)	8 (27.6%)	
Marital status	Yes	18 (64.3%)	21 (72.4%)	0.631
	No	10 (35.7%)	8 (27.6%)	
Smoking	Yes	3 (10.7%)	6 (20.7%)	0.302
	No	25 (89.3%)	23 (79.3%)	
Hookah use	Yes	7 (25.0%)	5 (17.2%)	0.473
	No	21 (75.0%)	24 (82.8%)	
Alcohol use	Yes	6 (21.4%)	4 (13.8%)	0.449
	No	22 (78.6%)	25 (86.2%)	
Physical activity level (MET‐m/d)	Baseline	3.11 ± 39.34	3.65 ± 38.59	0.491

*Note:* Values are means ± SDs for continuous variables and percentages for categorical variables. Independent sample *t*‐test for quantitative variables and *χ*2 test for qualitative variables.

The inflammatory biomarkers that were present in the curcumin and placebo groups at the beginning and the conclusion of the intervention are shown in Table 2. Additionally, the changes that occurred in these biomarkers are also shown. A substantial rise in hs‐CRP was seen in the control group, with a value of 1.53 ± 3.33 mg/L (*p* = 0.305). On the other hand, the intervention group had a drop that was not statistically significant. Furthermore, it is worth noting that the intervention group had a significant reduction in levels of IL‐6 (mean change: 5.28 ± 9.76, *p* = 0.009) after the intervention period. After accounting for baseline levels, the supplement's efficacy in reducing hs‐CRP was statistically significant. Regarding the impact of curcumin supplementation on NF‐κB levels in patients with HT by comparing the intervention and placebo groups, the placebo group showed a slight increase in NF‐κB levels (0.65 ± 0.16 to 0.68 ± 0.13), while the curcumin group exhibited a significant reduction (−0.09 ± 0.22 vs. 0.03 ± 0.15, *p* = 0.019) (Figure 2).

**TABLE 2:** The status of inflammatory markers of Hashimoto's patients in the intervention and control groups.

Variable	Study groups	Research time			*p*‐value	*p* for supplement effectiveness^a^
		Start of study	End of study	Mean changes		
hs‐CRP (mg/L)	Curcumin Group	3.51 ± 4.32	2.63 ± 3.34	−1.17 ± 5.70	0.019	**0.009**
	Placebo Group	3.40 ± 3.42	4.94 ± 4.00	1.53 ± 3.33	0.305	
	*p*‐value	**0.912**		**0.034**		
IL‐6 (pg/mL)	Curcumin Group	12.57 ± 8.91	7.38 ± 2.30	−5.28 ± 9.75	0.009	**0.271**
	Placebo Group	10.65 ± 6.35	8.70 ± 5.86	−1.94 ± 7.89	0.194	
	*p*‐value	**0.351**		**0.164**		
NF‐kB (pg/mL)	Curcumin Group	0.71 ± 0.18	0.63 ± 0.15	−0.09 ± 0.22	0.038	**0.051**
	Placebo Group	0.65 ± 0.16	0.68 ± 0.13	0.03 ± 0.15	0.293	
	*p*‐value	**0.226**		**0.019**		

Abbreviations: hs‐CRP, high‐sensitivity C‐reactive protein; IL‐6, interleukin‐6; NF‐κB, nuclear factor kappa B.

^a^ ANCOVA models were applied to compare post‐intervention outcomes between groups, adjusting for baseline values of hs‐CRP, IL‐6, and NF‐κB, as well as age, sex, BMI, and levothyroxine use.

## 4 Discussion

**FIGURE 3:**
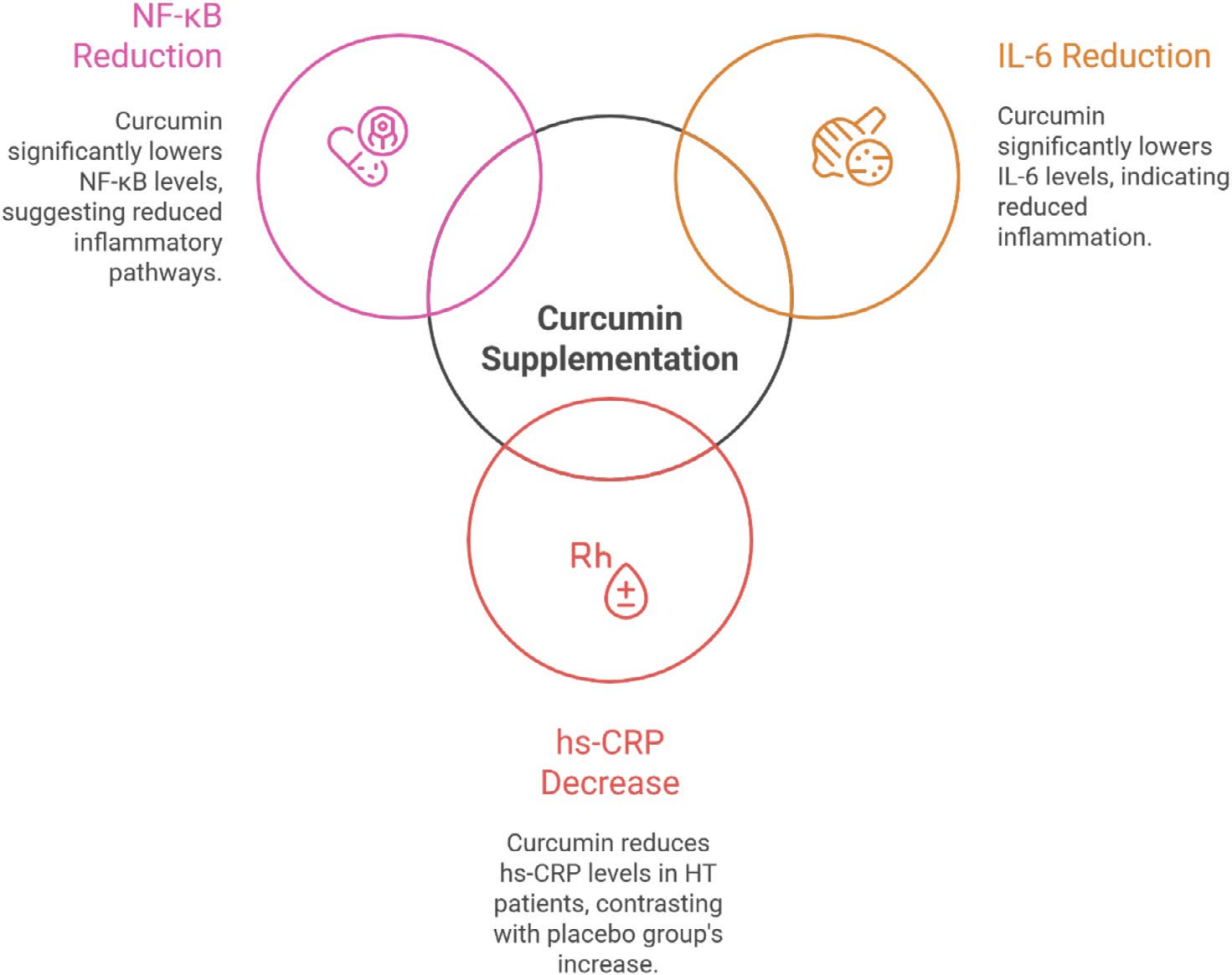
Anti‐inflammatory effects of curcumin in Hashimoto's thyroiditis.

To the best of our knowledge, this study represents the first clinical trial investigating the combined effects of an anti‐inflammatory diet and curcumin supplementation on patients with HT. The intervention significantly improved the level of inflammatory indices such as hs‐CRP, IL‐6, and NF‐κB levels in patients with HT (Figure 3). Unlike prior studies that examined curcumin supplementation or dietary interventions separately, our trial is the first to investigate the combined effects of curcumin supplementation and adherence to an anti‐inflammatory diet in patients with Hashimoto's thyroiditis. This dual approach strengthens the novelty of our work and provides new evidence on the potential synergistic benefits of nutritional and phytochemical strategies in autoimmune thyroid disease.

Curcumin is considered the key active compound in turmeric (Abd El‐Hack et al. 2021), and previous studies have shown its anti‐inflammatory (Pourhabibi‐Zarandi et al. 2022), antioxidant (Kaur et al. 2024; Ak and Gülçin 2008), antimicrobial (Hussain et al. 2022), antitumor (Ojo et al. 2022; Barcelos et al. 2022), and immune‐regulating effects (Allegra et al. 2022), with therapeutic potential in neurodegenerative (Tang and Taghibiglou 2017), cardiovascular (Li et al. 2020), and cerebrovascular diseases (Pu et al. 2013). Our intervention had considerable anti‐inflammatory benefits, shown by decreases in indicators such as hs‐CRP and IL‐6. In line with the findings of the present study, Ebrahimzadeh et al. performed a meta‐analysis to evaluate the effects of curcumin supplementation on inflammatory markers, namely CRP and ESR, in individuals with rheumatoid arthritis and ulcerative colitis. Their analysis revealed that administering curcumin in doses ranging from 250 to 1500 mg/day over a period of 8 to 12 weeks significantly reduced CRP levels compared to control groups (Ebrahimzadeh et al. 2021). Similarly, another comprehensive review and meta‐analysis of 15 randomized controlled trials (RCTs) evaluating the impact of curcumin supplementation on inflammation and oxidative stress biomarkers revealed that curcumin substantially decreased levels of IL‐6, hs‐CRP, and malondialdehyde (MDA). Nonetheless, no notable impacts were observed on TNF‐α or superoxide dismutase (SOD) levels. (Tabrizi et al. 2019). A review of 66 RCTs by Dehzad et al. evaluated the effects of turmeric and curcumin on inflammatory and oxidative balance in the body, revealing that supplementation significantly reduced inflammatory markers such as CRP, TNF‐α, and IL‐6, though no significant change was observed in IL‐1β. Additionally, curcumin improved antioxidant status by increasing TAC and SOD activity and decreasing MDA levels (Dehzad et al. 2023). Furthermore, curcumin treatment in thyroid cancer cell lines enhanced the expression of redifferentiation markers (TG and NIS), induced G2/M cell cycle arrest and apoptosis, and significantly downregulated NF‐κB p65 activity, resulting in decreased tumor progression (Schwertheim et al. 2017).

Although the precise mechanism of curcumin's action in HT is not fully understood, several potential pathways might have a role. Curcumin can exert substantial anti‐inflammatory effects by downregulating the NF‐κB pathway, thereby reducing the expression of pro‐inflammatory cytokines such as TNF‐α, IL‐1β, and IFN‐γ, which are implicated in thyroid cell damage (Buhrmann et al. 2011). In addition, curcumin suppresses cyclooxygenase‐2 (COX‐2) and inducible nitric oxide synthase (iNOS), further reducing inflammation. Its antioxidant properties also mitigate oxidative stress, a key factor in HT, by scavenging reactive oxygen species (ROS) and reducing lipid peroxidation (Lee et al. 2020). Additionally, curcumin's antioxidant properties could mitigate oxidative stress—a key factor in HT—by scavenging reactive oxygen species (ROS) and reducing lipid peroxidation (Priyadarsini et al. 2003).

Beyond its anti‐inflammatory and antioxidant roles, curcumin may regulate apoptosis and cell survival pathways. It can induce cell death in autoreactive immune cells while preserving thyroid cells through modulation of Akt, PI3K, and ERK signaling (Shakeri et al. 2022). Curcumin also influences mitochondrial membrane potential and regulates apoptotic proteins such as Bcl 2, thereby contributing to immune balance and thyroid cell protection. An in vitro investigation utilizing four assays related to the inflammatory features of arthritis demonstrated that curcumin markedly reduced neutrophil activation, angiogenesis, and synoviocyte proliferation. Importantly, it also significantly downregulated the expression of matrix metalloproteinases in chondrocytes, indicating curcumin's potential role in modulating key pathways involved in joint inflammation and degradation (Jackson et al. 2006).

Our study has several strengths, including a randomized controlled trial design, which ensured a balanced allocation of participants between intervention and control groups, reducing selection bias. The use of double‐blinding and placebo‐controlled capsules enhanced the reliability of the findings by minimizing performance and detection bias. Comprehensive outcome measurements covering thyroid‐related markers, inflammatory biomarkers, lipid profiles, anthropometric data, and nutrient intake provided a holistic evaluation of curcumin's effects. Additionally, adherence was closely monitored through regular follow‐ups, capsule counts, and phone calls, ensuring high compliance. Clear inclusion and exclusion criteria minimized confounding variables, strengthening the validity of the observed effects.

On the other hand, there are a few significant limitations. There is a possibility that the results cannot be generalized to larger or more varied populations due to the relatively small sample size, despite the fact that the sample size is statistically appropriate. The 12‐week duration may not be sufficient to observe long‐term effects of curcumin supplementation, particularly for chronic conditions like HT. Limited control over participants' adherence to the prescribed anti‐inflammatory diet introduced potential variability in dietary effects. Significant reductions in nutrient intake, especially in the control group, could have influenced some biochemical and inflammatory outcomes. These limitations should be addressed in future studies by incorporating a broader and more diverse sample size to improve generalizability and investigate the long‐term effects of curcumin supplementation with extended follow‐ups. Enhanced dietary control, such as providing standardized meals, could reduce variability. Investigating the mechanistic pathways of curcumin's effects on thyroid function and inflammation would provide deeper insights. Comparative studies with other anti‐inflammatory interventions like selenium or vitamin D would help evaluate relative efficacy. Expanding biomarker analyses to include oxidative stress markers or gut microbiota composition could yield a more comprehensive understanding of curcumin's impact. Larger and longer trials are required to confirm these effects and to explore underlying mechanisms in greater detail.

## 5 Conclusion

This 12‐week randomized, placebo‐controlled trial suggests that curcumin supplementation, when combined with an anti‐inflammatory diet, may help reduce inflammatory markers in patients with Hashimoto's thyroiditis. However, these results should be interpreted with caution given the sample size and study duration, and confirmation in larger, long‐term trials is warranted.

## Author Contributions

**Golbon Sohrab:** formal analysis.

## Funding

This research was financially supported by the National Nutrition and Food Technology Research Institute, affiliated with Shahid Beheshti University of Medical Sciences (Code 43006472). It is important to clarify that the funding organization had no influence on any part of the research process.

## Ethics Statement

This study was conducted in compliance with established ethical principles, and the research protocol received approval from the Ethics Committee of Shahid Beheshti University of Medical Sciences, Tehran, Iran (IR.SBMU.NNFTRI.REC.1402.035). Voluntary, written informed permission was acquired from all individuals before enrollment. Additionally, the trial was officially registered in the Clinical Trials Database under the identification number NCT05975866, with the registration submitted on August 8, 2023, ensuring transparency and adherence to recognized clinical research standards.

## Consent

This section is not applicable to the present study, as no individual patient data or identifiable information was included that would necessitate explicit consent for publication.

## Conflicts of Interest

The authors declare the absence of any actual or potential conflicts of interest, whether financial or otherwise, that may have affected the outcomes or interpretation of the results presented in this manuscript.

## Data Availability

The data that support the findings of this study are available from the corresponding author upon reasonable request.
